# The risk factors and impact of subchorionic hematoma in the first trimester in IVF twin pregnancies: a prospective cohort study

**DOI:** 10.3389/fmed.2023.1187344

**Published:** 2023-06-28

**Authors:** Youwen Mei, Yonghong Lin, Xiaoli Guo, Yangping Zhang, Fang Wang

**Affiliations:** Chengdu Women's and Children's Central Hospital, School of Medicine, University of Electronic Science and Technology of China, Chengdu, China

**Keywords:** *in vitro* fertilization, pregnancy loss, subchorionic hematoma, twin pregnancies, risk factors

## Abstract

**Objective:**

This study aimed to identify the risk factors for subchorionic hematoma (SCH) in the first trimester of *in vitro* fertilization (IVF) twin pregnancies and investigate the impact of SCH on pregnancy outcomes.

**Study design:**

A prospective cohort study was conducted at Chengdu Women and Children's Central Hospital. The study recruited patients who were identified with twin pregnancies in the first trimester, undergoing IVF treatment from January 2020 to May 2021. The demographic characteristics and pregnancy outcomes were compared between the SCH and the non-SCH groups. A logistic regression analysis was used to determine the risk factors for SCH and adverse pregnancy outcomes.

**Results:**

In the first trimester, 38% of patients developed SCH. The independent risk factors for SCH included male factor, hydrosalpinx, polycystic ovary syndrome (PCOS), previous miscarriage, and adenomyosis. With respect to the pregnancy outcomes, only the rate of twin pregnancy loss before 20 gestational weeks was significantly higher in the SCH group than in the non-SCH group. After adjusting for the confounding factors, the presence of SCH diminished the ovarian reserve, and previous miscarriage was independently related to twin pregnancy loss before 20 gestational weeks.

**Conclusion:**

This may be the first study to evaluate the risk factors of SCH in twin pregnancies who underwent IVF-ET/FET treatment, which may provide some theoretical basis for clinical practice in the future. Furthermore, it was found that the occurrence of SCH was associated with the loss of both pregnancies before 20 gestational weeks. Therefore, these patients should be offered increased surveillance and timely treatment.

## Introduction

Sub-chorionic hematoma (SCH), a collection of blood between the chorionic membrane and the uterine wall, is a common ultrasonic feature in the first trimester (1). The incidence of SCH varies from 1 to 48% in singleton pregnancy (2, 3). In recent years, SCH has become increasingly common in twin pregnancies, and many twin pregnancies were the result of *in vitro* fertilization (IVF) treatment (4). According to reports, IVF treatment was associated with an increased incidence of SCH in the first trimester (5). It was believed that SCH was caused by a partial detachment of the chorion from the uterine wall (6). However, the underlying causes of SCH in IVF twin pregnancies are unclear. In singleton pregnancy, SCH was observed to be associated with adverse pregnancy outcomes such as spontaneous abortion, preterm birth, placental abruption, and fetal growth restriction (2, 7). In twin pregnancies, the presence of SCH was stated to be the independent risk factor for the loss of one or both fetuses before 20 weeks of gestation (8) but was unrelated to pregnancy outcomes >24 weeks (9). However, limited data differentiate between IVF and non-IVF twin pregnancies. Therefore, to provide evidence for clinical application, we conducted this study to identify the risk factors for SCH and the impact of SCH in IVF twin pregnancies.

## Materials and methods

This was a longitudinal analysis of data prospectively collected in Chengdu Women's and Children's Central Hospital. Women who had two gestational sacs at the first-trimester ultrasound after IVF treatment from January 2020 to May 2021 were included in this study. We excluded patients with fetal abnormalities, elective termination of pregnancy, twin-to-twin transfusion syndrome, monoamniotic twins, results of multifetal pregnancy reduction, and who were lost to follow-up.

In our practice, all women had their initial ultrasound at 6^0/7^-6^7/7^ weeks, and they had ultrasound examinations every 2 weeks. We collected the data of each ultrasound report for the occurrence and size of an SCH. The demographic and baseline clinical information was collected from the patients.

**Figure 1:**
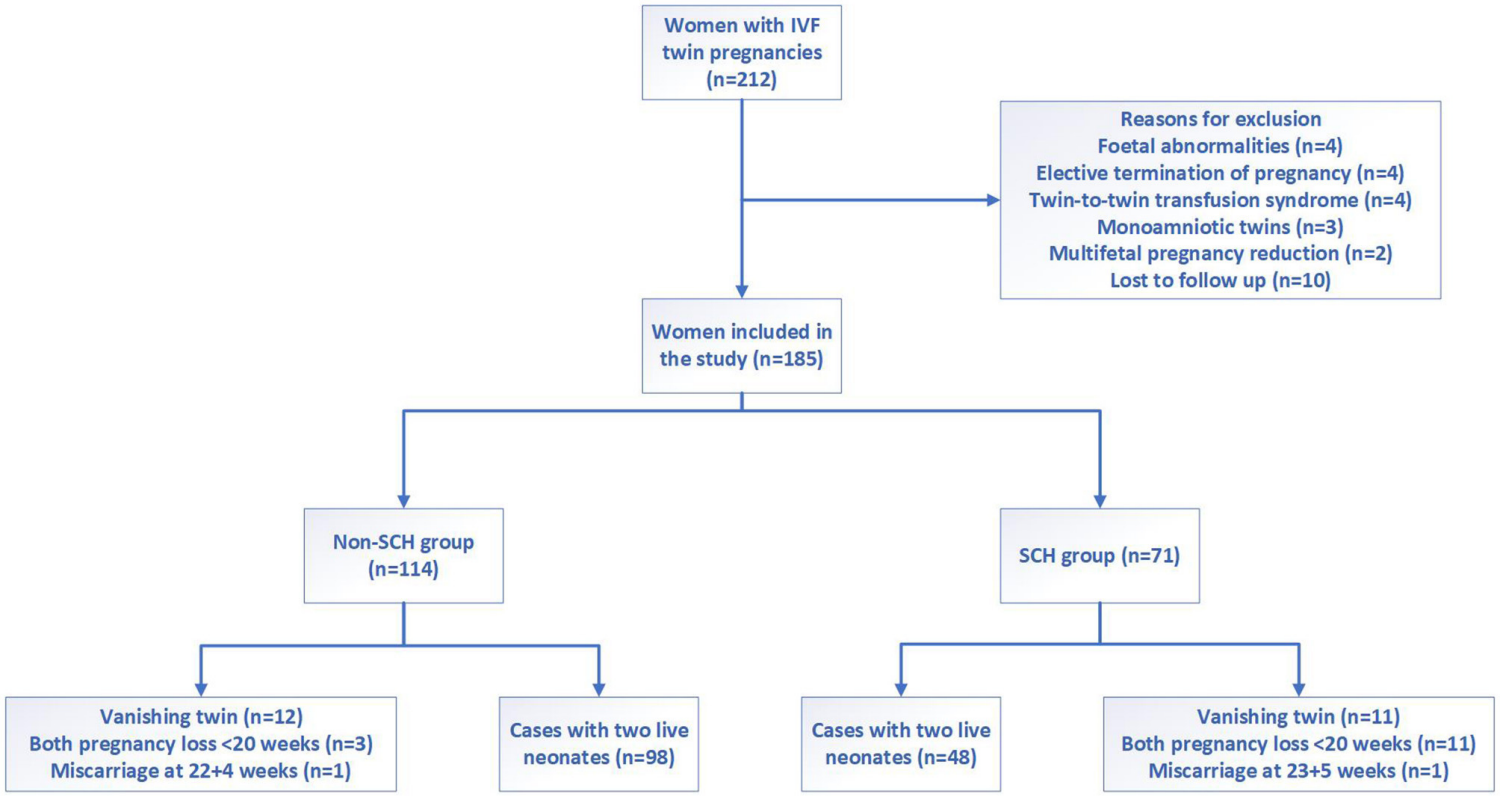
Flowchart showing the study selection of women with IVF twin pregnancies.

On ultrasound imaging, SCH was identified as the fluid that accumulated behind the chorionic membrane. The mean diameter of length and width was used to determine the SCH size (Figure 1). The gestational age was determined by the embryo transfer time. The cause of infertility included hydrosalpinx, fallopian tube obstruction, polycystic ovary syndrome (PCOS), adenomyosis, diminished ovarian reserve (DOR, anti-Müllerian hormone level < 1.1 ng/mL or total antral follicle count ≤ 6), male factor (oligospermia, asthenospermia, and teratozoospermia), and unexplained cause. In our study, patients with hydrosalpinx underwent tubal ligation or salpingectomy before embryo transfer. The IVF-related factors included embryo stage (cleavage and blastocyst), gonadotropin (Gn) dose, number of retrieved oocytes, inner cell mass (ICM) stage, trophectoderm stage, infertility type (primary and secondary), type of embryo transfer (fresh embryo transfer and frozen-thawed embryo transfer), frozen–thawed transfer cycle (natural cycle and hormone replacement cycle), and endometrial thickness (EMT) at embryo transfer.

The pregnancy outcome measures included vanishing twin syndrome (one fetus ceased development), twin pregnancy loss before 20 gestational weeks, spontaneous preterm delivery (< 37 weeks), preterm premature rupture of membranes < 37 weeks (PPROM), hypertensive disorder, gestational diabetes mellitus (GDM), intrahepatic cholestasis of pregnancy (ICP), selective intrauterine growth restriction (sIUGR), placental abruption, hypothyroidism, fetal distress, neonatal birth weight, and asphyxia neonatorum.

Baseline data and pregnancy outcomes were compared between the SCH group and the non-SCH group, and the independent risk factors for SCH and adverse pregnancy outcomes were identified. This study was approved by the Ethics Committee of Chengdu Women's and Children's Central Hospital. Written informed consent was obtained from all the participants.

### Statistical analysis

Statistical analysis was performed using the SPSS 19.0 software. Categorical variables were assessed using a chi-square test, and continuous variables were evaluated by Student's *t*-test or the Mann–Whitney *U*-test according to the data distribution. Potential confounding factors were added to multivariable logistic regression analysis. In the regression analysis, a “forward: conditional” method was performed for variables that differed in the non-SCH group and the SCH group (*P* < 0.1) in the univariate analysis. The relationship between the size and emerging time of SCH and adverse pregnancy outcomes was determined using Pearson's correlation analysis.

## Results

### Baseline characteristic

**Figure 2:**
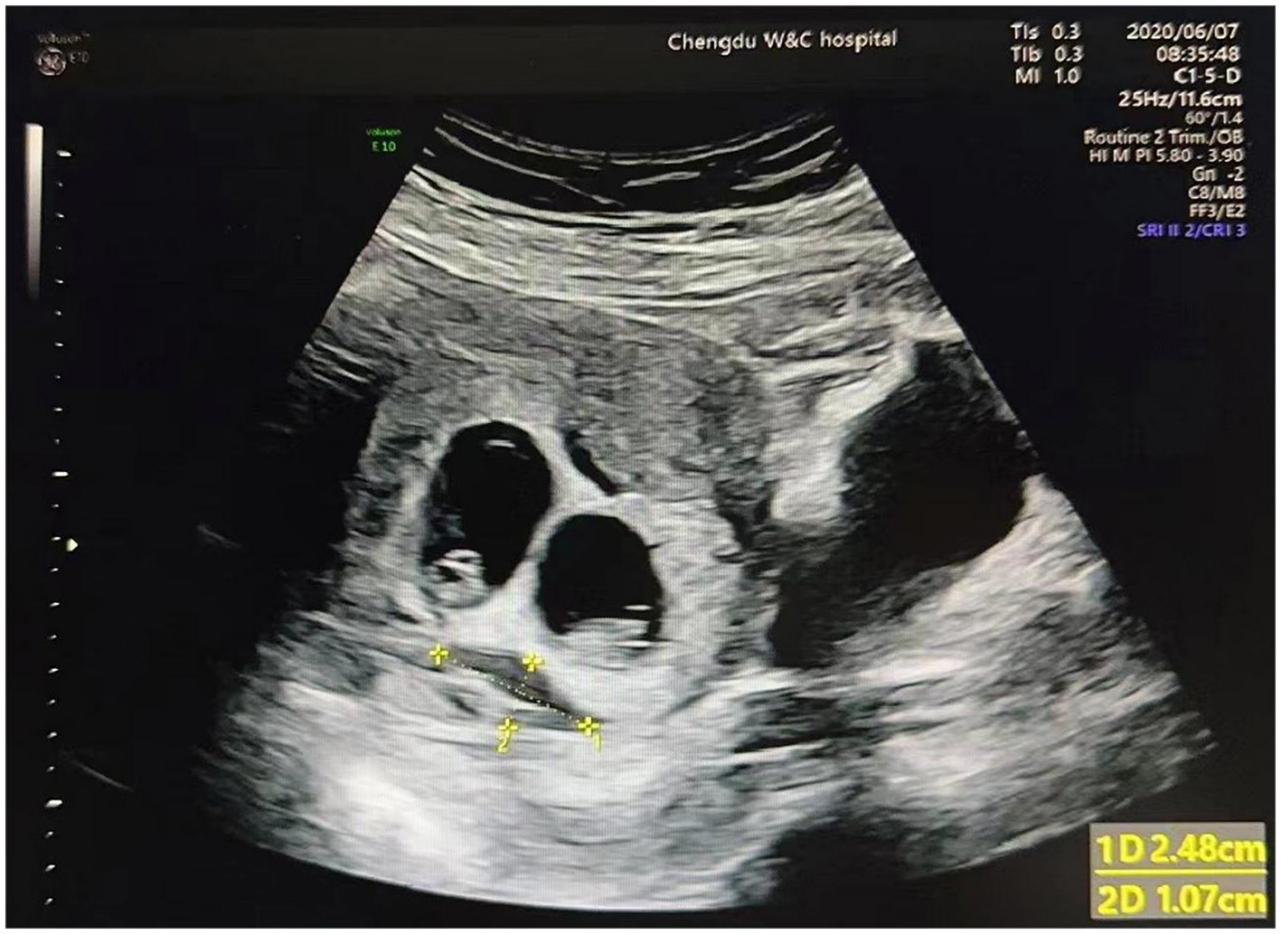
Ultrasound image for SCH in twin pregnancies.

A total of 212 IVF twin pregnancy cases were recruited, and 185 cases were included for the final analysis in our study. Among them, 38% (71/185) of pregnancies developed SCH in the first trimester (Figure 2).

The comparison of baseline data revealed that the rate of previous miscarriage was higher in the SCH group than in the non-SCH group. In addition, the cause of infertility was different in the two groups. The rate of hydrosalpinx, adenomyosis, and male factor was significantly higher in the SCH group than in the non-SCH group. In contrast, the rate of fallopian tube obstruction was significantly lower in the SCH group than in the non-SCH group. There was no significant difference between the two groups in terms of patient's age, body mass index (BMI), gravity, parity, previous preterm birth, infertility type, DOR rate, unexplained infertility rate, the total dose of Gn, number of retrieved oocytes, embryo stage, ICM grade, trophectoderm grade, embryo transfer type, frozen embryo transfer protocol, EMT, LMWH/aspirin use, and progesterone level at embryo transfer (Table 1). We used a logistic regression analysis to determine which factor was independently related to SCH. The results revealed that the independent risk factors for SCH in IVF twin pregnancies were previous miscarriage, hydrosalpinx, PCOS, adenomyosis, and male factor (**Table 3**).

**Table 1:** Baseline characteristics between the non-subchorionic hematoma (SCH) group and the SCH group.

	**Non-SCH (*n =* 114)**	**SCH (*n =* 71)**	***p^*a*^***
Age, years	30.8 ± 3.5	31 ± 3.9	0.724
BMI, kg/m^2^	21.3 (19.8–24)	21.6 (19.7 + 23)	0.813
Gravity	3 (2, 3)	3 (2, 3)	0.502
Parity	0 (0–0)	0 (0–0)	0.641
Prior preterm labor times	7 (6.1%)	3 (4.2%)	0.744^b^
Previous miscarriage rate^*^	3 (2.6%)	7 (9.9%)	0.046^b^
Infertile duration	3 (2, 3, 4, 5)	3 (2, 3, 4, 5)	0.432
MCDA	7 (6.1%)	2 (2.8%)	0.307
DCDA	107 (93.9%)	69 (97.2%)	0.307
Hydrosalpinx^*^	3 (2.6%)	13 (18.3%)	0.001^b^
Fallopian tube obstruction^*^	99 (86.8%)	36 (50.7%)	< 0.0001
PCOS^*^	2 (1.8%)	6 (8.5%)	0.056^b^
Adenomyosis^*^	0	6 (8.5%)	0.032^b^
DOR	11 (9.6%)	7 (9.9%)	0.963
Male factor^*^	2 (1.8%)	12 (16.9%)	< 0.0001^b^
Unexplained	3 (2.6%)	5 (7%)	0.263^b^
Cleavage	60 (52.6%)	41 (57.7%)	0.497
Blastocyst	54 (47.4%)	30 (42.3%)	0.497
Dose of gonadotropin	2,647.1 ± 941	2,500 ± 814	0.277
Number of retrieved oocytes	16.9 ± 8.4	18.5 ± 9.5	0.24
ICM A/B	46 (88.5%)	26 (86.7%)	0.811
ICM C	6 (11.5%)	4 (13.3%)	1^b^
Trophectoderm A/B	41 (78.8%)	19 (63.3%)	0.127
Trophectoderm C	11 (21.2%)	11 (36.7%)	0.127
Primary infertility	56 (49.1%)	29 (40.8%)	0.272
Secondary infertility	58 (50.9%)	42 (59.2)	0.272
Fresh embryo transfer	14 (12.3%)	5 (7%)	0.254
Frozen-thawed embryo transfer	100 (87.7%)	66 (93%)	0.254
Natural cycle	74 (74%)	45 (68.2%)	0.415
Hormone replacement cycle	26 (26%)	21 (31.8%)	0.415
Endometrium thickness (mm)	10 (9, 10, 11, 12)	10 (9–12.5)	0.7
Progesterone level within 2 weeks	23 (18.6–31)	22.6 (18.6–29.7)	0.935
Aspirin use	37 (32.5%)	19 (26.8%)	0.412
LMWH use	64 (56.1%)	41 (57.7%)	0.83

^*^P < 0.05, MCDA, monochorionic diamniotic; DCDA, dichorionic Diamniotic; PCOS, polycystic ovary syndrome; DOR, diminished ovarian reserve; ICM, inner cell mass. ^a^Chi-square or Student's *t*-test. ^b^Fisher's exact test.

### Pregnancy outcomes

The comparison of pregnancy outcomes revealed that twin pregnancy loss rate before 20 weeks was significantly higher in the SCH group than the non-SCH group (15.5 vs. 2.6%, *P* = 0.001). However, other pregnancy outcomes, such as vanishing twin, delivery mode, spontaneous preterm birth, PPROM, hypertensive disorder, ICP, GDM, sIUGR, fetal distress, placenta abruption, hypothyroidism, neonatal birth weight, and asphyxia neonatorum were comparable between the two groups (Table 2). The logistic regression analysis revealed that male factor, hydrosalpinx, PCOS, previous miscarriage, and adenomyosis were the independent risk factors of SCH (Table 3).

**Table 2:** Comparison of pregnancy outcomes between the non-subchorionic hematoma (SCH) group and the SCH group.

	**Non-SCH (*n =* 114)**	**SCH (*n =* 71)**	***p^*a*^***
Vanishing twin	12 (10.5%)	11 (15.5%)	0.319
Twin pregnancy loss < 20 weeks^*^	3 (2.6%)	11 (15.5%)	0.003^b^
Miscarriage between 20 and 24 weeks for cervical incompetence	1 (0.9%)	1 (1.4%)	0.622 ^b^
Cases with twin perinatal outcomes	Non-SCH (*n =* 98)	SCH (*n =* 48)	
Cesarean section	95 (96.9%)	44 (91.7%)	0.161
Vaginal delivery	3 (3.1%)	4 (8.3%)	0.218^b^
Spontaneous preterm birth	14 (14.3%)	10 (20.8%)	0.316
PPROM	11 (11.2%)	8 (16.7%)	0.359
Hypertensive disorder	6 (6.1%)	3 (6.25%)	1^b^
ICP	6 (6.1%)	2 (4.2%)	1^b^
GDM	7 (7.1%)	3 (6.25%)	1^b^
sIUGR	3 (3.1%)	2 (4.2%)	0.664^b^
Fetal distress	3 (3.1%)	3 (6.25%)	0.395^b^
Placenta abruption	2 (2%)	0	1^b^
Hypothyroidism	3 (3.1%)	0	0.551^b^
Asphyxia neonatorum	1 (1%)	0	1^b^
Birth weight of A neonate	2,500 (2,200–2,785)	2,430 (2,047.5–2,722.5)	0.119
Birth weight of B neonate	2,465 (2,100–2,700)	2,300 (1,962.5–2,300)	0.057

^*^p < 0.05, PPROM, preterm premature rupture of membranes; ICP, intrahepatic cholestasis of pregnancy; GDM, gestational diabetes mellitus; Siugr, selective intrauterine growth restriction. ^a^Chi-square or Student's *t*-test. ^b^Fisher's exact test.

**Table 3:** Adjusted risk of subchorionic hematoma (SCH) in *in vitro* fertilization (IVF) twin pregnancies.

**Factors**	**Regression coefficients (b)**	**Significance (*p*)**	**OR**	**OR (95% CI)**
Male factor	2.674	0.001	14.494	3.008–69.839
Hydrosalpinx	2.282	0.001	9.797	2.543–37.746
PCOS	2.241	0.008	9.399	1.81–48.821
Previous miscarriage	1.859	0.011	6.417	1.538–26.778
Adenomyosis	2.53	0.026	12.56	1.358–116.198

PCOS, polycystic ovary syndrome. The final regression model included previous miscarriage rate, hydrosalpinx, fallopian tube obstruction, PCOS, adenomyosis, and male factor.

The logistic regression analysis revealed that SCH, DOR, and previous miscarriage were the independent risk factors of twin pregnancy loss before 20 weeks (Table 4). To explore which SCH-related index was related to twin pregnancy loss before 20 weeks, we performed Pearson's correlation analysis. Unexpectedly, either SCH size (Pearson's correlation index −0.157, *P* = 0.203) or emerging time (Pearson's correlation index 0.106, *P* = 0.395) was associated with twin pregnancy loss before 20 weeks.

**Table 4:** Adjusted risk of twin pregnancy loss before 20 gestational weeks.

**Risk factors**	**Twin pregnancy loss < 20 weeks**	**Unadjusted OR (95% CI)**	**Adjusted OR (95% CI)**	***p***
Previous miscarriage^*^		6.39 (1.449–28.178)	5.452 (1.019–29.176)	0.048
Yes	21.40%			
No	4.10%			
Male factor		3.967 (0.963–16.334)	2.398 (0.466–12.329)	0.295
Yes	21.40%			
No	6.40%			
Fallopian tube obstruction		0.336 (0.111–1.012)	0.925 (0.248–3.459)	0.908
Yes	50%			
No	74.90%			
DOR^*^		4.486 (1.245–16.165)	5.004 (1.131–22.143)	0.034
Yes	28.60%			
No	8.20%			
SCH^*^		5.03 (1.137–22.246)	6.783 (1.822–25.258)	0.001
Yes	15.50%			
No	2.60%			

^*^P < 0.05, DOR, diminished ovarian reserve. The final regression model included subchorionic hematoma, previous term delivery, previous miscarriage, conceived methods, and accompanied bleeding.

## Discussion

Our study revealed that previous miscarriage, hydrosalpinx, PCOS, and male factor were all independent risk factors affecting the development of SCH, and the rate of twin pregnancy loss before 20 weeks increased significantly in patients with SCH.

In our study, the incidence of SCH in twin pregnancies reached 38%, which was higher than in previous studies (8, 9). The reason may be that the patients who underwent IVF treatment had an initial ultrasound in an earlier gestational week (during the 6^th^ to 7^th^ weeks). Therefore, more SCH cases could be found before the hematoma was absorbed in non-IVF cases.

The presence of SCH may be related to embryo quality and endometrial receptivity. In our investigation, the male factor (poor sperm quality) was the independent risk factor for SCH. The reason may be that sperm affected genomic material transfer and the transportation of sperm-derived factors which are essential for early embryonic development (10). Our research also revealed that hydrosalpinx remained a risk factor for SCH even after tubal ligation or salpingectomy. This may be owing to the possibility that the immune inflammatory environment associated with hydrosalpinx can disrupt ovarian and endometrial functions, further affecting embryo quality and endometrial receptivity (3). It is well-known that PCOS is related to disordered endometrial receptivity, which presents as dysregulation of endometrial sex hormone receptors, increased endometrial insulin resistance, immune-inflammatory disorder, and altered uterine vascularity (11). Therefore, patients with PCOS have a higher chance of developing SCH. Similarly, adenomyosis may increase the risk of SCH by causing hypoxia (12) and altering endometrial vascularization and epithelial-mesenchymal transition/mesenchymal–epithelial transition (13). Some adverse factors related to embryo quality and endometrial receptivity may still be present in the current pregnancy for women who have experienced previous miscarriages. Therefore, it may be reasonable to say that previous miscarriage was the independent risk factor SCH.

In terms of the pregnancy outcomes, our study revealed that SCH was an independent risk factor of twin pregnancy loss before 20 weeks. This was consistent with previous studies. Tuuli's meta-analysis revealed that the presence of SCH increased the risk of pregnancy loss by 2-fold in non-IVF pregnancies (14). In addition, Ji et al. study stated that the presence of SCH was related to the loss of both fetuses before 20 weeks of gestation in women pregnant with twins (8). However, SCH was not related to vanishing twins in our study which was in contrast with Ji et al. study. The reason may be that the mechanism of vanishing twins was related to chromosomal abnormality (15). In our analysis, apart from the loss of both fetuses, SCH was not associated with other pregnancy outcomes. Similarly, Yue reported that SCH had no impact on the pregnancy outcomes of IVF pregnancies, including gestational weeks, preterm birth, and neonatal birthweight (3). Ji et al. (8) and Naqvi et al. (9) further reported that SCH was not associated with adverse pregnancy outcomes such as stillbirth, preeclampsia, preterm labor, low birth weight, postpartum hemorrhage, and fetal distress in twin pregnancies. It was observed that the impact of SCH appeared to be “all” or “nothing” in IVF twin pregnancies. The underlying mechanism may be the shallow trophoblast invasion and impaired angiogenesis related to SCH and secondary mechanical effects of the hematoma (14).

Unexpectedly, our study revealed that the emerging time of SCH was not related to twin pregnancy loss before 20 weeks. In contrast to a previous study, which reported that the earlier an SCH was identified, the higher the rate of subsequent pregnancy failure (16). The reason may be that in this study, all patients had an ultrasound at approximately 6–7 gestational weeks. However, in non-IVF patients, they may have initial ultrasound later if they did not experience vaginal bleeding or abdominal pain. Regarding the SCH diameter, our study was consistent with Ji et al. study (8), which reported no association between SCH volume and largest diameter and pregnancy loss before 20 weeks of gestation. It may be owing to the irregular shape of uterine hematomas, which made accurate measurement difficult. Furthermore, different measurement methods could also affect the measurement results (16).

This study may be the first to prospectively identify the risk factors for SCH and the impact of SCH on pregnancy outcomes in IVF twin pregnancies. All patients had an ultrasound at 6–7 weeks in this study; therefore, selection bias was reduced. However, there were some limitations. First, our study had a relatively small sample size which may reduce its statistical power. Second, it was uncertain whether the persistence of SCH would affect the pregnancy outcomes given that the time interval of ultrasound varied among patients.

## Conclusion

The independent risk factors for SCH in women who had IVF twin pregnancies included previous miscarriage, hydrosalpinx, PCOS, adenomyosis, and male factor. Additionally, SCH may increase the rate of twin pregnancy loss before 20 weeks of gestation. However, either SCH emerging time or size was related to miscarriage before 20 weeks of gestation. Large prospective randomized studies are required to determine the risk factors for SCH and its effects on IVF twin pregnancies.

## Data availability statement

The original contributions presented in the study are included in the article/supplementary material, further inquiries can be directed to the corresponding author.

## Ethics statement

The studies involving human participants were reviewed and approved by Chengdu Women and Children's Central Hospital. The patients/participants provided their written informed consent to participate in this study. Written informed consent was obtained from the individual(s) for the publication of any potentially identifiable images or data included in this article.

## Author contributions

YM and YL drafted the manuscript and participated in data collection and analysis. FW participated in the design of the study and performed the statistical analysis. XG and YZ participated in data collection and analysis. All authors contributed to the article and approved the submitted version.
